# Criminal Justice-Involved Veterans Not Engaged in Primary Care in the Veterans Health Administration

**DOI:** 10.1177/21501319251348134

**Published:** 2025-06-17

**Authors:** Jack Tsai, Austin Lampros, Sean Clark, Anne Dunn, Thomas P. O’Toole

**Affiliations:** 1 Homeless Programs Office, U.S. Department of Veterans Affairs (VA) Central Office, Washington, DC, USA; 2 Department of Management, Policy, and Community Health, University of Texas Health Science Center at Houston (UTHealth) School of Public Health, Houston, TX, USA; 3 Office of the Undersecretary for Health- Clinical Operations, Washington, DC, USA; 4 Warren Alpert Medical School of Brown University, Providence, RI, USA

**Keywords:** primary care, veterans, behavioral health, criminal justice involvement

## Abstract

**Introduction:**

Many adults involved in the criminal justice system have various healthcare needs. The Veterans Health Administration (VHA) operates programs to connect criminal justice-involved veterans to healthcare, including primary care. This study examined veterans in the Veterans Justice Programs (VJP) to understand which veterans are not empaneled in VA primary care and their associated characteristics and service use.

**Methods:**

A retrospective cohort study was conducted with 20 395 veterans who participated in VJP in 2023. Data on sociodemographic characteristics, clinical status, primary care empanelment, and service utilization from VHA medical records were analyzed.

**Results:**

Across the country, about 88% of veterans in VJP were empaneled in primary care who attended a mean of 3.63 (SD = 4.63) primary care visits over 12 months compared to a mean of 0.18 (SD = 0.54) primary care visits among veterans not empaneled. Bivariate analyses found that empaneled veterans in VJP were significantly less likely to have any mental health or substance use disorder than non-empaneled veterans. However, multivariable analyses revealed the characteristics most strongly and significantly associated with empanelment in primary care among VJP veterans were use of outpatient medical care (aOR = 16.53, 95% CI = 9.32-31.43), use of outpatient mental health/substance use treatment (aOR = 1.83, 95% CI = 1.27-2.70), military sexual trauma (aOR = 1.66, 95% CI = 1.35-2.06), and being non-Hispanic black (aOR = 1.61, 95% CI = 1.35-1.96 compared to Hispanic black or white).

**Conclusions:**

Empanelment in primary care is associated with use of behavioral healthcare among criminal justice-involved veterans indicating opportunities for integrated care initiatives in VHA facilities.

## Introduction

Many studies have reported that adults involved in the criminal justice system tend to have higher rates of various medical problems than other adults, including sexually transmitted diseases, pancreatitis, hepatitis, and substance use disorders.^1,2^ Criminal justice involvement can be defined as interaction with the criminal justice system, including pretrial detention, conviction, incarceration, probation, and parole.^3^ Depending on which aspect of the criminal justice system they are involved in, criminal justice-involved adults have reported various barriers to accessing healthcare and social services, including lack of healthcare coverage or other payment issues, stigma or perceived prejudice regarding criminal justice involvement, and other internal and external issues with navigating care.^4-7^

Military veterans are an important segment of adults involved in the criminal justice system in the United States given their service to the country, military training, and documented social adjustment issues after leaving military service.^8-10^ The United States Department of Veterans Affairs (VA) consists of the Veterans Health Administration (VHA) which operates medical centers and clinics across the country to provide a wide range of primary care and other healthcare services to veterans. VHA has 2 relatively new programs focused on providing care to criminal justice-involved veterans. These 2 programs, the Veterans Justice Outreach (VJO) and the Health Care for Re-Entry Veterans (HCRV) programs, are collectively referred to as the Veterans Justice Programs (VJP). Most veterans in VJP are eligible for a variety of VHA healthcare benefits and may not face the same eligibility or insurance barriers as non-veterans. So one of the main goals of VJP is to connect justice-involved veterans to VHA healthcare and community resources and a number of studies have been conducted documenting the healthcare needs of this population.^11,12^ It may also be worth noting that VJP is an important part of VA’s system-wide effort to end veteran homelessness and is organized within the VHA Homeless Programs Office given important intersections between criminal justice involvement and homelessness.^13,14^ As one of the identified areas in need of further research, there is a need to better understand how well justice-involved veterans in the VHA system are being connected to healthcare, including primary care services.^15^ Moreover, VHA has a stated mission to “honor America’s veterans by providing exceptional health care that improves their health and well-being” and strive to achieve this for all veterans.^16^

Thus, the objectives of the current study were to analyze national VHA data to answer several key questions: (1) How many veterans in VJP are empaneled in VA primary care nationally? and (2) What differences in sociodemographic and clinical characteristics are there between VJP veterans who are and are not empaneled in VA primary care? Empanelment in primary care refers to the process of assigning patients to specific primary care providers or care teams within a healthcare system. Empanelment is an indicator of an established patient-provider relationship and is foundational for ensuring continuity, efficiency, and quality of care.^17^ Various studies have found that patients who are not empaneled use primary care are more likely to experience discontinuous, and fragmented care due to not having assigned providers.^18,19^

## Methods

Data were extracted from the VHA’s Corporate Data Warehouse (CDW) and Homeless Operations and Evaluation System (HOMES) on a total of 20 395 veterans in VJP in calendar year 2023. CDW includes data from veterans’ medical records^20^ and HOMES includes data on veterans’ encounters with VHA homeless programs.^21^ The first recorded visit in CDW or HOMES in 2023 for each veteran served as that veteran’s index date. Of the total study sample, veterans empaneled in VHA primary care were identified as being empaneled with a start date prior to 12/31/2023 and no end date or an end date after 12/31/2022. The VHA Office of Primary Care uses the Primary Care Management Module (PCMM) to track panel assignment of veterans for empanelment. There is a PCMM coordinator at each VHA medical center whose responsibility is to assign veterans to a patient aligned care team for primary care prior to their first appointment and no later than 24 h from the creation of the patient’s first appointment, per VHA Directive 1406(1). Veterans who have had no primary care appointments for 3 years are removed from their panel. Thus, all veterans receiving primary care should technically be empaneled, but there may be cases in which veterans have primary care visits recorded in their medical records but are not considered empaneled because they are “pending” empanelment (due to the PCMM coordinator), they were empaneled before or after the observation period, or they were receiving specialized care (e.g., infectious disease clinic, renal care) that may not necessarily require empanelment. Duplicate data were limited to the most recent data; and veterans who died prior to 2023, had extreme age values, or had an empanelment that expired were excluded.

Individual characteristics of veterans selected for this study was guided by the Andersen healthcare utilization model,^22^ which conceptualizes predisposing variables, enabling variables, and need variables as driving healthcare access and use. In this study, predisposing variables, including demographics such as age, sex, and race. Enabling variables included variables, such as geographic region and VA service-connected disability. Need variables included variables, such as psychiatric diagnoses and chronic medical conditions.

Predisposing and enabling variables were extracted from the VHA CDW. In terms of need variables, diagnoses of psychiatric conditions were extracted from CDW for the period within 1 year before the veterans’ index date. Psychiatric diagnoses were based on International Classification of Diseases, Tenth Revision (ICD-10) codes and the major mental health disorders (schizophrenia, bipolar disorder, major depression, other mood disorder, posttraumatic stress disorder, other stress disorder, generalized anxiety disorder, other anxiety disorder, other mental health disorder) as well as substance use disorders (alcohol use disorder, cocaine use disorder, opioid use disorder, amphetamine use disorder, hallucinogen use disorder, sedative use disorder, inhalant use disorder, other psychoactive substance use disorder). Also using ICD-10 codes, the Charlson Comorbidity Index (CCI) was used as a measure of medical comorbidity, which consists of 17 different medical conditions that are weighted for a total score.^23^

Data on types of VHA healthcare utilization were extracted based on the time-period within 1 year after veterans’ index date. Data on healthcare use included VHA outpatient medical care, outpatient mental health/substance use treatment, inpatient medical care, inpatient mental health/substance use treatment, and emergency department services. Medical care included both primary care and specialty care; in some analyses primary care was specifically examined.

### Data Analysis

Among the sample of VJP veterans, bivariate analyses were conducted to compare those empaneled and not empaneled in primary care using chi-square tests and independent t-tests. Cohen’s *d* and difference (Δ) in percentages were also calculated for effect sizes. Then logistic regression analyses were conducted to compare empaneled and non-empaneled VJP veterans including only statistically significant variables with a group difference of Cohen’s d ≥ 0.20 or ≥5% in the bivariate analyses. Before logistic regressions were conducted, any variables with high multicollinearity with others were removed from the model. Investigative plots, Q-Q plots, calculation of variance of inflation factors, and goodness-of-fit tests were also conducted to check there were no violations of assumptions for all tests. Variables with any multi-collinearity (variable clustering >0.70) or variance inflation factor (VIF) greater than or equal to 0.10 were excluded from the model. There were no significant model fit issues as evidenced by the Omnibus goodness-of-fit test. Adjusted odds ratios (aORs) with 95% Confidence Intervals (CIs) were calculated for effect sizes.

## Results

In the total sample of veterans in VJP (n = 20 395), 17 874 (87.64%) were empaneled in primary care and 2521 (12.36%) were not. As shown in Table 1, about 87% to 88% of veterans in VJP were empaneled in primary care across the 5 major geographic regions of the country with little variability by region. Of veterans who had any primary care visits, VJP veterans empaneled in primary care attended a mean of 3.63 (SD = 4.63) primary care visits in 12 months; by comparison, VJP veterans not empaneled in primary care attended a mean of 0.18 (SD = 0.54) primary care visits in 12 months. Of all VJP veterans, primary care visits per veteran were low across geographic regions when veterans who had no primary care visits were included in the total denominator (Table 1). By geographic region, empaneled VJP veterans in the Pacific region had the greatest total average number of primary care visits per veteran.

**Table 1.** Proportion of Veterans in VJP Empaneled in Primary Care, 2023.

Region	# of veterans empaneled in primary care (% empaneled among all veterans in VJP)	# of outpatient primary care visits in 2023 among empaneled VJP veterans	# of outpatient primary care visit per empaneled VJP veteran
Continental	2660 (87.67)	1005	0.38
Midwest	4617 (87.10)	1795	0.39
North Atlantic	3849 (88.50)	1607	0.42
Pacific	3939 (87.17)	2507	0.64
Southeast	2809 (88.00)	1202	0.43

As shown in Table 2, VJP veterans empaneled in primary care were significantly more likely to be female, married, have a VA service-connected disability, report combat exposure, and to be non-Hispanic black or white than VJP veterans not empaneled in primary care. In terms of clinical characteristics, empaneled VJP veterans were significantly less likely to be diagnosed with any mental health or substance use disorder than non-empaneled VJP veterans and they generally had similar rates of diagnoses across individual disorders (none >∆5.0%). Not surprisingly, VJP veterans were significantly more likely to use any outpatient medical care, emergency department, and inpatient medical care but were less likely to use any outpatient or inpatient mental health/substance use treatment.

**Table 2.** Bivariate Comparison of Individual Characteristics Among Veterans in VJP Who Were Empaneled and Not Empaneled in Primary Care, 2023.

Variable	Empaneled in primary care (n = 17 874)	Not empaneled in primary care (n = 2521)	Test of difference t or χ^2^	∆% or Cohen’s *d*
	Mean/Count (SD/%)	Mean/Count (SD/%)		
Sociodemographics				
Age	48.13 (SD = 13.58)	48.35 (SD = 14.46)	0.4724	*d* = 0.02
Race/ethnicity				
Non-Hispanic white	9719 (54.38%)	1190 (47.20%)	<.001***	7.17%
Non-Hispanic black	3894 (21.79%)	371 (14.72%)		7.07%
Hispanic black or white	1803 (10.09%)	253 (10.04%)		0.05%
Mixed race/other	738 (4.13%)	90 (3.57%)		0.56%
Missing	1720 (9.62%)	617 (24.47%)		−14.85%
Sex				
Male	16674 (93.29%)	2417 (95.91%)	<.001***	−2.63%
Female	1200 (6.71%)	103 (4.09%)		2.63%
Marital status				
Married	4698 (26.28%)	545 (21.62%)	<.001***	4.67%
Single/never married	5364 (30.01%)	758 (30.07%)		−0.06%
Divorced/separated	7167 (40.10%)	911 (36.14%)		3.96%
Widowed	357 (2.00%)	77 (3.05%)		−1.06%
Missing or unknown	288 (1.61%)	230 (9.12%)		−7.51%
Geographic region				
Continental	2660 (14.88%)	374 (14.84%)	0.2176	0.05%
Midwest	4617 (25.83%)	684 (27.13%)		−1.30%
North Atlantic	3849 (21.53%)	500 (19.83%)		1.70%
Pacific	3939 (22.04%)	580 (23.01%)		−0.97%
Southeast	2809 (15.72%)	383 (15.19%)		0.52%
VA service-connected disability	57.73 (SD = 40.37)	19.79 (SD = 32.99)	<.001***	d = 0.96
Combat exposure	512 (2.86%)	65 (2.58%)	0.455	0.29%
Military sexual trauma	1877 (10.50%)	107 (4.24%)	<.001***	6.26%
Clinical characteristics				
Any mental health disorder	1861 (14.47%)	27 (16.98%)	<.001***	−2.51%
Any substance use disorder	1588 (12.35%)	26 (16.35%)	<.001***	−4.00%
Schizophrenia	195 (1.52%)	2 (1.26%)	<.001***	0.26%
Other psychosis	12 (0.09%)	0 (0.00%)	<.001***	0.09%
Bipolar spectrum disorder	344 (2.68%)	7 (4.40%)	<.001***	−1.73%
Major depression	943 (7.33%)	8 (5.03%)	<.001***	2.30%
Other mood disorder	782 (6.08%)	8 (5.03%)	<.001***	1.05%
PTSD	990 (7.70%)	14 (8.81%)	<.001***	−1.10%
Other stress disorder	488 (3.80%)	9 (5.66%)	<.001***	−1.86%
GAD	279 (2.17%)	3 (1.89%)	<.001***	0.28%
Other anxiety disorder	771 (6.00%)	5 (3.14%)	<.001***	2.85%
Other mental health disorder	501 (3.90%)	3 (1.89%)	<.001***	2.01%
Cannabis use disorder	683 (5.31%)	5 (3.14%)	<.001***	2.17%
Cocaine use disorder	481 (3.74%)	4 (2.52%)	<.001***	1.23%
Alcohol use disorder	1125 (8.75%)	20 (12.58%)	<.001***	−3.83%
Opioid use disorder	317 (2.47%)	3 (1.89%)	<.001***	0.58%
Amphetamine use disorder	618 (4.81%)	8 (5.03%)	<.001***	−0.22%
Hallucinogen use disorder	26 (0.20%)	0 (0.00%)	<.001***	0.20%
Sedative use disorder	66 (0.51%)	0 (0.00%)	<.001***	0.51%
Inhalant use disorder	4 (0.03%)	0 (0.00%)	0.0455*	0.03%
Other psychoactive substance use disorder	442 (3.44%)	4 (2.52%)	<.001***	0.92%
Any suicidality	334 (2.60%)	3 (1.89%)	<.001***	0.71%
Dementia	7 (0.05%)	0 (0.00%)	0.0082**	0.05%
Charlson Comorbidity Index	0.81 (SD = 1.12)	0.81 (SD = 1.03)	0.9425	d = 0
Health service use				
Any outpatient medical care visits	1854 (30.43%)	14 (17.28%)	<.001***	13.14%
Any outpatient mental health/substance use treatment visits	1923 (31.56%)	42 (51.85%)	<.001***	−20.29%
Any emergency department visits	1644 (26.98%)	19 (23.46%)	<.001***	3.52%
Any inpatient medical care days	388 (6.37%)	2 (2.47%)	<.001***	3.90%
Any inpatient mental health/substance use treatment days	284 (4.66%)	4 (4.94%)	<.001***	−0.28%

Abbreviations: SD, standard deviation; PTSD, posttraumatic stress disorder; GAD, generalized anxiety disorder.

*** *P* < .001.

These bivariate findings were followed with multivariable analyses (Table 3) that found VJP veterans empaneled in primary care were significantly more likely to have a VHA service-connected disability (aOR = 1.03), to be non-Hispanic black (aOR = 1.61 compared to Hispanic black or white), divorced/separated (aOR = 1.20 compared to married), to report military sexual trauma (aOR = 1.66), and to use any outpatient medical care (aOR = 16.53) or outpatient mental health/substance use treatment (aOR = 1.83) compared to non-empaneled VJP veterans.

**Table 3.** Multiple Logistic Regression Comparing Individual Characteristics of VJP Veterans Empaneled and Not Empaneled in Primary Care, 2023.

Variable	Adjusted odds ratio	95% CI
VA service-connected disability	1.03	(1.03, 1.03)***
Race/ethnicity (Ref: Non-Hispanic black)		
Hispanic black or white	0.62	(0.51, 0.74)***
Missing	0.37	(0.31, 0.43)***
Mixed race/other	0.79	(0.61, 1.03)
Non-Hispanic white	0.89	(0.78, 1.01)
Marital status (ref: divorced/separated)		
Married	0.83	(0.74, 0.94)**
Missing or unknown	0.25	(0.2, 0.32)***
Single/never married	0.84	(0.75, 0.94)**
Widowed	0.69	(0.52, 0.91)**
Military sexual trauma	1.66	(1.35, 2.06)***
Any outpatient medical care visits	16.53	(9.32, 31.43)***
Any outpatient mental health/substance use treatment visits	1.83	(1.27, 2.70)**

** *P* < .05. *** *P*< .001.

## Discussion

While the majority of veterans in VJP were empaneled in VHA primary care, over one-tenth of VJP veterans were not empaneled, indicating opportunities for increased and targeted outreach of these veterans in primary care. In particular, VJP veterans who were not empaneled tended to have certain demographic profiles (male, Hispanic, unmarried) and were not as connected with VA benefits or healthcare as empaneled veterans. There may be institutional and cultural reasons influencing empanelment that deserve further study, such as notions of masculinity among male veterans that deter help-seeking,^24^ historical distrust of healthcare providers among Hispanic individuals,^25^ and stigma related to criminal justice involvement.^26^ These reasons may also interact with each other, and the Andersen healthcare utilization model,^22^ which we used as a framework for the analyses, may also be helpful in hypothesizing these institutional and cultural factors as predisposing and enabling variables that need to be incorporated in future research. Regardless, our findings suggest better engaging veterans overall in VA may facilitate a higher probability of empanelment in VA primary care. VA has made great efforts to connect veterans to VHA care through marketing (e.g., airing public service announcements), enacting policies (e.g., expanding eligibility), and conducting outreach (e.g., through VJP and other VA programs).^27-29^ The VHA’s large integrated healthcare system can facilitate various avenues for service integration and care linkages for veterans once they are enrolled.

While VJP veterans empaneled in primary care were generally less likely to have any mental health or substance use disorder diagnoses than non-empaneled veterans, it was notable that use of any outpatient mental health/substance use treatment was associated with 83% higher odds of being empaneled in VHA primary care after controlling for sociodemographic and clinical characteristics. Therefore, this finding suggests a notable paradox, which is that veterans with behavioral health issues may be less likely to be empaneled even though they may have greater needs given their higher rates of medical comorbidity;^30,31^ but veterans who are participating in behavioral healthcare in VA may be more likely to be empaneled because of overall connection to VA care overall and may have more opportunities to be involved in various healthcare services. We cannot infer causation with our study, but presumably better connecting veterans to behavioral healthcare may facilitate greater opportunities for empanelment in primary care and vice versa. This paradox then indicates the potential for integrated primary care and behavioral health services in VHA to benefit veterans in both domains.^32-34^

In order to overcome this paradox, issues of fragmented care may need to be addressed which can be complicated with veterans are dual users of VA and community providers, and with issues like court-mandated treatment. VHA has implemented integrated behavioral healthcare systemwide for many years with some evidence of its success as well as challenges;^34-36^ however, this work has also revealed there is a need for more focus on specific subgroups or situations (e.g., rural veterans). Veterans in VJP may particularly benefit from integrated behavioral healthcare given their rates of physical and mental health disorders,^11,37-39^ and so specific efforts to empanel this subgroup may have multiple positive downstream effects.

Several limitations should be mentioned. First, we only examined empanelment and use of VA primary care and other healthcare services. Some veterans enrolled in VA healthcare also use other community care which we did not examine and we did not have data on non-empaneled veterans not in the VA healthcare system. We used a broad definition of empanelment in VA, and some operational analyses may use more restrictive definitions of empanelment or VJP veterans. Second, given the retrospective cohort design, no causality between variables can be inferred. Third, our analyses relied on VA administrative data and accurate documentation by PCMM on empanelment status. Fourth, our study sample only included veterans participating in VJP and so further study is needed to determine whether our findings generalizable to other samples of criminal justice-involved veterans and non-veterans.

In conclusion, about 1 of 10 veterans in VJP are not empaneled in primary care in the VA’s integrated healthcare system. Since use of VA behavioral healthcare was associated with primary care empanelment, integrating primary care and behavioral health approaches may be well suited in the VA healthcare system to benefit criminal justice-involved veterans.
